# One year later: Highlighting the challenges and opportunities in disseminating a breathing-retraining digital behaviour change intervention

**DOI:** 10.1177/2055207620936441

**Published:** 2020-07-14

**Authors:** Ben Ainsworth, Anne Bruton, Mike Thomas, Lucy Yardley

**Affiliations:** 1 Department of Psychology, University of Bath, UK; 2 NIHR Biomedical Research Centre, Faculty of Medicine, University of Southampton, UK; 3 School of Health Sciences, University of Southampton, UK; 4 Primary Care and Population Sciences, University of Southampton, UK; 5 School of Psychological Science, University of Bristol, UK; 6 School of Psychology, University of Southampton, UK

**Keywords:** Digital behaviour change interventions, dissemination, implementation, asthma

## Abstract

Digital behaviour change interventions can provide effective and cost-effective treatments for a range of health conditions. However, after rigorous evaluation, there still remain challenges to disseminating and implementing evidence-based interventions that can hinder their effectiveness ‘in the real world’. We conducted a large-scale randomised controlled trial of self-guided breathing retraining, which we then disseminated freely as a digital intervention. Here we share our experience of this process after one year, highlighting the opportunities that digital health interventions can offer alongside the challenges that must be addressed in order to harness their effectiveness. Whilst such treatments can support many individuals at extremely low cost, careful dissemination strategies should be proactively planned in order to ensure such opportunities are maximised and interventions remain up to date in a fast-moving digital landscape.

## Introduction

The BREATHE (Breathing Retraining: A Trial of Home Exercise) randomised controlled trial (RCT) in 655 primary care patients demonstrated that self-guided breathing retraining was an effective and cost-effective way to improve quality of life for adults with asthma.^1^ Our digital intervention research group subsequently offered free online access to the intervention for people with asthma and healthcare professionals. Recent key recommendations for increasing behavioural science research uptake in public health practice have highlighted the need to understand real-world practicalities and identify barriers and opportunities relevant to implementation.^2^ Here we share our experience of disseminating and implementing the online version of the intervention from the BREATHE trial.

### What is the breathing retraining intervention?

Physiotherapy breathing retraining for asthma is a non-pharmacological intervention that aims to ameliorate dysfunctional breathing patterns that can impair quality of life in patients with asthma, with demonstrable benefits for patient well-being.^3,4^ Breathing retraining is recommended in asthma management guidelines (British Thoracic Society, National Institute for Health and Care Excellence). However, there are currently insufficient National Health Service (NHS) physiotherapists with appropriate skills to provide face-to-face breathing retraining for all those who might benefit, so alternative delivery methods are needed. The BREATHE trial found that digital self-guided breathing retraining (using a DVD and booklet (DVDB)) delivered equivalent benefits to a face-to-face intervention,^1^ and was superior to ‘usual care’, with a similar effect size to the stepping-up of pharmacological therapies.^5^ The DVDB intervention (‘Breathing Freely’) was meticulously constructed using the person-based approach – an iterative development approach with detailed qualitative research involving healthcare professionals and patients.^6^ The DVDB contained technique demonstrations, motivational components, educational content and behaviour-change support components such as practice planning.

### How was the breathing retraining intervention adapted for online dissemination?

When the BREATHE trial was initially designed, DVD was a widely adopted technology and an effective way to implement a self-management intervention for a widely prevalent chronic condition.^7^ However, in the perpetually developing field of digital health, the lengthy timescale of RCTs means that specific intervention delivery methods used during trials may no longer be appropriate for wide-scale implementation;^8^ by the time findings from the BREATHE trial were published, the DVD was no longer ubiquitous and most digital audio-visual content was streamed digitally to computers or mobile devices in both medical and entertainment settings.^9^ Digital behaviour change interventions (DCBIs) have the potential to improve health by providing effective, convenient and cost-effective interventions to impact a range of behaviours and health conditions.^10^ By providing remote access to complex treatments, they are both adaptive and scalable, and are likely to be increasingly relevant in the current healthcare climate that emphasises the role of preventative individual self-management.^11^

Therefore, the DVDB intervention was adapted to create an online web-based intervention (called ‘Breathing Freely Online’), in line with current technology, to be available freely online (http://www.breathestudy.co.uk). Two tailored versions were created: i) for patients, including all intervention content, and ii) for healthcare professionals (with additional information about the trial, access to the patient version and access to a ‘demo’, which did not require registration). Both versions were disseminated within the primary research publication^1^ as well as through other avenues (such as press releases, social media, blogs).

Breathing Freely Online was developed using the LifeGuide software,^12^ which allows the development and modification of web-based interventions without the need for specific programming knowledge. The adaptation took less than one week to develop, with minimal support costs (see Table 1).

**Table 1.** Costs involved in adapting the DVDB intervention to a web-based format for one year.

Role	Cost
Developer expertise and time	£1500
Ongoing support delivered by LifeGuide team (per annum)	£500
Additional costs (e.g. website domain name)	£20
Total	£2020

### How was ‘Breathing Freely Online’ used over 12 months?

A particular advantage of DCBIs is the ability to monitor usage through digital metrics – for example, how many people visited the website, registered and viewed key pages – which can provide insight about intervention engagement. The key usage metrics are presented in Table 2.

**Table 2.** Usage metrics of ‘Breathing Freely Online’ during the first year.

Role	Cost
Users signed up to full ‘Breathing Freely Online’ intervention	456
Those who read core information or watched at least one video	392
Users who used HCP demo intervention (sign-up not required)	1099
Those who read core information or watched at least one video	872

Breathing Freely Online had over 1500 users and – alongside results from the RCT – is likely to have proved cost-effective on a population level. Such metrics must always, however, be interpreted with caution. Recent work by our group has emphasised the significant variability in effective engagement^13,14^ – that is, the engagement with interventions needed to achieve sufficient behavioural change to affect outcomes (some patients might need to use Breathing Freely Online many times before changing their breathing habits, whilst others might notice a benefit immediately).

The DVDB intervention was formally qualitatively evaluated during the main trial, with 29 people with asthma completing semi-structured ‘think-aloud’ interviews in which they reported their experiences of using the materials.^4^ We have also received feedback from online users, both HCPs and patients, who contacted the research team after using the intervention. In Table 3, we have reported some purposively selected examples of some of this feedback.

**Table 3.** Feedback from users of Breathing Freely Online.

User	Detailed feedback
Liz Hore (LH: publican, UK)	A patient who was diagnosed with asthma 40 years previously, commented that while Breathing Freely Online would never replace pharmacological treatments for asthma, it offered other benefits. In particular, LH noted that the digital intervention was particularly useful as respiratory physiotherapy was hard to access. LH also did not continue to access content but rather remembered the techniques that were recommended; however, she noted that it was useful to have in order to ‘jog the memory’.
Anna Boniface (AB: Respiratory Physiotherapist, UK)	AB saw Breathing Freely Online as a valuable tool to support patients who were identified as having dysfunctional breathing patterns. She noted that it provided a useful adjunct to patient self-management plans. As well as the videos, AB frequently used the interactive motivational content that identified dysfunctional breathing with patients and suggested techniques to address it. AB also noted that the website could support respiratory physiotherapists who had limited time and contact with patients.
James Dodd (JD: Consultant in Respiratory Medicine, UK)	JD commented that while evidence for the benefits of breathing retraining for asthma has existed for some time, low accessibility within the NHS has meant that clinicians were typically unable to offer it. JD said that an evidence-based breathing retraining intervention was very easy to access and, therefore, likely to be recommended to patients.

Note: LH, AB and JD were all willing to be named for this publication and have approved this feedback, which was not formally analysed.

DCBIs such as Breathing Freely Online are unlikely to be acceptable for all patients with asthma, given the considerable disease heterogeneity and individual variability in personal preferences. Alongside positive comments, we also received negative feedback from patients – for example, some commented that they would have much rather had a physiotherapist to correct them if they were breathing with a poor technique, and that the website needed more additional information. However, we consider DCBIs to be ‘self-selecting’ in that patients who find them acceptable will use them and potentially take benefit from them, while others who view them less favourably will continue with their existing treatment. By using the person-based approach, we aimed to maximise the number of patients for whom the intervention would be acceptable.

### What have we learnt in the last year? Highlighting challenges and opportunities

We have learnt much from offering a free intervention after the full RCT, and recommend it as an effective and cost-effective method to translate clinical science into patient benefit. We will finish this communication by highlighting the key challenges and opportunities from our experience.

#### 1. Opportunities afforded by extremely low cost

We were able to enable access for over 1500 users at a very low cost – most of which was the initial adaptation from the DVDB format. This cost-effectiveness (which was supported by data from the BREATHE trial) will only increase as the scale increases, with more users and no additional per-person costs. We acknowledge that there are some associated costs. For example, it is desirable to provide ongoing technical support to address technical barriers to access and usability that may arise due to changes in servers, browsers or devices. While we were able to provide this through *our internal web-support (‘LifeGuide’) team*,^12^ not all intervention developers will be able to use existing infrastructure. Therefore, we encourage all prospective developers to plan their dissemination strategies carefully. For example, relevant charitable bodies, industrial partners or other stakeholders may provide the necessary support for intervention dissemination – but may need to be involved right at the start of the development process in order to provide this.

#### 2. The challenge of promotion: raising awareness of evidence-based medicine

Recent calls have highlighted the difficulty in raising awareness of ‘evidence-based’ digital interventions, particularly differentiating effective treatments from commercial opportunism.^15^ Depending on the nature of the DCBI, there are several different areas to target efforts to raise awareness, such as amongst healthcare professionals (e.g. clinical newsletters), existing patient resources (e.g. NHS Direct) and broader approaches such as search engine optimization. The BREATHE RCT was published in a high-profile peer-reviewed journal, and we worked with Asthma UK and several press outlets in order to maximise patient awareness of Breathing Freely Online, alongside social media and other academic outlets. However, we may have benefited from specific expertise focusing on implementation in areas that could have further improved effectiveness, such as awareness amongst GPs. We recommend planning such efforts during the development process, to co-ordinate press coverage and maximise patient awareness.

#### 3. The need to remain ‘up to date’ in a digital landscape

While the existing version of Breathing Freely Online (http://www.breathestudy.co.uk) was designed for current technology, it is well known that digital interventions must be frequently updated to remain compatible with new technology and to take advantage of new functionality.^8^ However, care must be taken during the updating of any empirically evidenced intervention to ensure that it remains evidenced. For example, when we initially adapted Breathing Freely Online from the DVDB intervention, additional components and functionality were considered (such as revised video content, interactive quizzes). However, it was felt that changing the initial content would compromise its ‘evidence-based’ nature by removing it too far from the DVDB intervention that was evaluated in the RCT. To ensure that DCBIs are continually updated for current technology while remaining evidence-based, we encourage key stakeholders and developers to identify ‘core intervention content’ that is fundamental to the intervention. Such core content is highly specific to each intervention and must be carefully defined. Examples of core content could include specific behaviour change techniques, particular images or pictures that resonate with users, or information delivery formats such as weekly notifications. This allows developers to consider whether future interventions of the intervention contain core content, and is particularly important if relying on external partners for dissemination, to ensure that any adaptations remain evidence-based without needing additional trial evaluation.

## Conclusion

Our dissemination of Breathing Freely Online was effective and cost-effective, but has highlighted valuable lessons for prospective intervention development and evaluation teams. We particularly encourage i) working with stakeholders to produce proactive dissemination plans that ensure that evidence-based digital products remain up to date, and ii) using a range of methods to increase patient awareness and ensure that the potential benefits of DCBIs are maximised.
